# Spatial analysis of paraneoplastic cerebellar degeneration in ovarian cancer with anti-Yo syndrome and SCA1

**DOI:** 10.1007/s00415-026-13884-0

**Published:** 2026-07-27

**Authors:** Tamara Künzle, Lucas Rincon de la Rosa, Clément Vialatte de Pémille, Alice Leprince-Laurenge, Alberto Picca, Giulia Coarelli, Delphine Leclercq, Alexandra Dürr, Dimitri Psimaras, Agusti Alentorn

**Affiliations:** 1 ICM, INSERM U 1127, CNRS UMR 7225, UMRS 1127, Paris Brain Institute, Sorbonne University, Paris, France; 2 Neurological Department, Groupe Hospitalier Paris Saint-Joseph, Paris, France; 3 Université Paris Cité, Paris, France; 4 Charles Foix, DMU Neurosciences, Service de Neuro-Oncologie-Institut de Neurologie, AP-HP, Hôpitaux Universitaires La Pitié Salpêtrière, Paris, France; 5 Department of Neuroradiology, APHP, La Pitié-Salpêtrière Hospital, Sorbonne University, F-75013 Paris, France; 6 APHP-Salpêtrière Hospital, DMU BioGem, CNRS, INSERM, Paris Brain Institute, Sorbonne University, Paris, France

**Keywords:** Neurological paraneoplastic syndrome, Cerebellar ataxia, Cerebellar atrophy, Cortical thickness and connectivity

## Abstract

**Background:**

Anti-Yo paraneoplastic cerebellar degeneration (PCD) is a rare autoimmune disorder linked to ovarian and breast cancers. Neurological symptoms often precede cancer diagnosis, yet conventional imaging techniques may fail to detect early cerebellar changes. This study quantitatively assessed cerebellar atrophy and network alterations in anti-Yo PCD patients compared to healthy controls and patients with spinocerebellar ataxia type 1 (SCA1).

**Methods:**

We analyzed structural MRI data from 11 antiYo PCD patients, 17 healthy controls, and 17 SCA1 patients. Cerebellar lobular segmentation and cortical thickness measurements were conducted. Structural covariance networks were built using inter-lobular Pearson correlation coefficients (threshold |*r*| > 0.5), with graph theory metrics assessing connectivity. Univariate and age-adjusted multivariate analyses evaluated group differences, and machine learning assessed the discriminative power of regional morphometric measures.

**Results:**

AntiYo PCD patients showed pronounced anterior cortical thinning, while SCA1 atrophy was milder and more posterior. Two PCD subtypes emerged: one with severe atrophy, another with nearnormal thickness. Network analysis revealed increased node strength and clustering coefficients, but reduced betweenness centrality in PCD, suggesting altered network hierarchy and widespread clustering that may reflect pathological reorganization. In cross-validated analysis, regional cerebellar features distinguished PCD, SCA1, and controls with promising AUC values.

**Conclusions:**

Anti-Yo PCD is characterized by anterior cerebellar vulnerability and network reorganization distinct from SCA1. These morphometric and connectivity markers are candidate imaging biomarkers for early diagnosis and subgroup stratification in paraneoplastic cerebellar degeneration.

**Supplementary Information:**

The online version contains supplementary material available at 10.1007/s00415-026-13884-0.

## Introduction

Paraneoplastic cerebellar degeneration (PCD) is a rare but severe paraneoplastic syndrome characterized by rapid onset of cerebellar ataxia and other neurological deficits. Unlike direct tumor invasion or metastasis, PCD arises from the indirect effects of cancer, typically mediated by an autoimmune response. This syndrome is most commonly associated with malignancies, such as ovarian cancer and breast cancer [1]. The underlying mechanism involves tumors that express antigens normally restricted to the cerebellum, which inadvertently triggers an immune response against cerebellar neurons, particularly Purkinje cells. Among the various antibodies implicated in PCD, the anti-Yo antibody, also known as the anti-Purkinje cell cytoplasmic antibody 1 (PCA-1), is one of the most frequently diagnosed in clinical settings [1]. Anti-Yo-mediated PCD predominantly occurs in women, correlating with its strong association with breast and ovarian cancers [2].

A better understanding of PCD is particularly important because approximately 80% of affected patients exhibit neurological symptoms prior to their cancer diagnosis [2]. Early recognition of PCD can facilitate prompt diagnosis and treatment of the underlying malignancy, potentially improving patient outcomes by stopping the progression of both oncological and neurological disease. Despite its clinical significance, the pathophysiology of PCD remains incompletely understood. Conventional cerebellar magnetic resonance imaging (MRI) often appears normal, especially early in the disease, obscuring the relationship between imaging findings and the underlying cerebellar pathology [3]. Advanced neuroimaging techniques have begun to shed light on the subtle structural changes that occur before visible atrophy is detectable. Recent studies employing quantitative structural MRI, including voxel-based morphometry and region of interest analysis, have demonstrated that cerebellar atrophy in PCD patients may follow distinct patterns compared to other cerebellar disorders.

Adult-onset degenerative cerebellar ataxias, or spinocerebellar ataxias (SCAs), represent a group of hereditary neurodegenerative disorders characterized by progressive ataxia resulting from cerebellar and spinal cord degeneration. Among these, spinocerebellar ataxia type 1 (SCA1) is one of the most common autosomal dominant inherited polyglutamine disorders [4]. Comparing cerebellar atrophy profiles between anti-Yo-positive PCD patients and SCA1 patients can elucidate disease-specific patterns of degeneration and potentially reveal unique biomarkers for differential diagnosis.

In this study, our objective was to quantitatively analyze cerebellar atrophy in anti-Yo-positive PCD patients and compare it with healthy controls and patients with SCA1.

Current state-of-the-art knowledge indicates that cerebellar atrophy in PCD is less well-characterized compared to other cerebellar disorders. Studies have shown variable results, with some reporting significant volume loss in specific cerebellar regions while others find more diffuse changes [3].

This study advances the quantitative analysis of cerebellar atrophy in anti-Yo PCD patients by employing a multifaceted neuroimaging approach. By comparing these patients to both SCA1 individuals and healthy controls, we identify unique and overlapping patterns of cerebellar degeneration, thereby enhancing our understanding of PCD’s neuropathological landscape and paving the way for improved diagnostic and therapeutic interventions.

## Methods

### Recruitment

Genetically confirmed SCA1 patients (*n* = 17) were recruited at the Reference Centre for Rare Diseases, Pitié-Salpêtrière Hospital (Paris) between November 2011 and December 2015 as part of the BIOSCA study (ClinicalTrials.gov NCT01470729; ID RCB 2010-A01324-35). The protocol was approved by the Comité de Protection des Personnes Île-de-France VI (AOM10094, Ref 105-10), and all participants provided written informed consent. Anti-Yo patients (*n* = 11) were enrolled at the same center from 2008 to 2017. In addition, healthy controls (*n* = 17) were selected retrospectively from the publicly available IXI dataset (EPSRC project “Information eXtraction from Images,” GR/S21533/02) [5]. The IXI dataset comprises fully anonymized MR images of neurologically normal volunteers acquired at three London hospitals on 1.5 T and 3 T scanners; its use is permitted under a CC BY-SA 3.0 license.

### Data acquisition

MRI acquisitions of SCA1 patients were performed on a 3 T whole-body Siemens MAGNETOM Trio scanner (Siemens Medical Solutions, Erlangen, Germany) using a standard Siemens transmit body coil and 32-channel receive head coil array. The MRI system was upgraded during the study period and hence twelve datasets were acquired on a 3 T whole-body Siemens MAGNETOM Prisma scanner (Siemens Medical Solutions, Erlangen, Germany). The rest of subjects participating in this study were acquired on a 3 Tesla MRI, the remainder on 1.5 T MRI machines (PREMIER, General Electric Healthcare). The median image acquisition criteria were: Repetition Time = 9.6, Echo Time = 4.6, Number of Phase Encoding Steps = 208, Echo Train Length = 208, Reconstruction Diameter = 240, Acquisition Matrix = 208 × 208, Flip Angle = 8.0 with sequence voxels size 1 × 1 mm and slice thickness 1 mm for T1-weighted images.

### Analysis

The T1-weighted images were used for subsequent analyses. Images from patients and controls were transformed into Neuroimaging Informatics Technology Initiative (NIfTI) format and then subjected to image normalization by N4 Bias Field Correction, to correct errors associated with the non-homogeneity due to the magnetic field, using the n4BiasFieldCorrect function of the package Advanced Normalization Tools in R (ANTsR) (v.0.5.6.1) [6]. Finally, the brain MRIs from this cohort were co-registered to a reference MRI (Montreal Neurological Institute, MNI152) using an Affine with deformable transformation using the symmetric normalization (SyN) (antsRegistration of the ANTsR package). The automated volumetric analysis for the brain was carried out from the NIfTI T1 files via CEREbellum Segmentation (CERES), which obtains a volumetric segmentation of the different lobules of the cerebellum [7, 8]. Quality control of the segmentation outputs was performed by visual inspection of each subject’s cerebellar parcellation overlaid on the native T1-weighted image; no subjects required exclusion on this basis. The same preprocessing and CERES segmentation pipeline was applied identically to the IXI control subjects and to the patient cohorts.

All analyses were performed using Python 3.11. NIfTI files were loaded and data extracted using the NiBabel library. The data were then resampled using the MNI152 template and plotted using the nilearn [9] library. Voxel-wise data points were mapped to 24 lobules (anterior lobules: Lobule I–II, Lobule III, Lobule IV, Lobule V; posterior lobules: Lobule VI, Lobule Crus I, Lobule Crus II, Lobule VIIB, Lobule VIIIA, Lobule VIIIB, Lobule IX, Lobule X; each lobule separated into left and right hemisphere) using a NiFTI template with labeled regions.

For each lobule, the median cortical thickness was calculated by aggregating values across all voxels assigned to that lobule using the region template. For heatmaps, correlation matrix analysis and clustering, lobule thicknesses were normalized using *Z*-scores.

Descriptive statistical data are presented as mean values with standard deviations. Univariate statistical analysis was performed using Welch’s *t* tests to compare the median cortical thickness between patient groups (anti-Yo PCD vs. control, anti-Yo PCD vs. SCA1, SCA1 vs. control). Resulting p values were corrected for multiple comparisons using the FDR method [10].

We performed linear regression for multivariate analysis, using the Ordinary Least Squares (OLS) method implemented in the python statsmodels [11] library. Patient group (anti-Yo PCD, SCA1 or control) and age were included as predictors for median lobule thickness. Time after diagnosis was excluded as a predictor due to its strong correlation with group membership (time after diagnosis *t*_*D*_ = 0 for all subjects in the control or SCA1 group). The p values corresponding to the beta coefficient of the group effect were extracted and subsequently FDR-corrected. For statistical analysis, a *p* value of *p <* 0*.*05 was regarded as significant. For network analysis, we used the python libraries NetworkX [12]. Network graphs were created using the structural covariance matrices, determining an edge between two lobules by a Pearson correlation coefficient of |*r*| *>* 0*.*5. To assess the robustness of the network findings to the choice of threshold, sensitivity analysis was performed by repeating the network-metric computation across a range of absolute thresholds (|*r*| > 0.3, 0.4, 0.5, 0.6, 0.7). In addition, a proportional thresholding approach was applied, retaining the top 10, 20, 30, 40, and 50% of the strongest connections to control for density differences across groups. Key metrics were compared across thresholds for all three groups. 3D node coordinates were determined by calculating the center of mass for each lobule.

Structural covariance (SC) matrices were obtained from each group. We computed node strength, betweenness centrality, (global) clustering coefficient and efficiency. Node strength is the sum of edge weights attached to a node. Betweenness centrality o*f* a node *v* is the sum of the fraction of all-pairs shortest paths that pass through *v*:

$$c_{B} \left( v \right) = \sum\limits_{s,t \in V} {\frac{{\sigma \left( {s,t\left| v \right.} \right)}}{{\sigma \left( {s,t} \right)}}}$$

where *V* is the set of nodes, *σ*(*s,t*) is the number of shortest (*s,t*)-paths, and *σ*(*s,t*|*v*) is the number of those paths passing through some node *v* other than *s,t*. If *s* = *t*, *σ*(*s,t*)= 1, and if *v* ∈ *s,t*, *σ*(*s,t*|*v*)= 0 [13]. The global clustering coefficient is defined as

$$C = \frac{1}{n}\sum\limits_{v \in G} {c_{v} }$$

where *n* is the number of nodes in *G* [14, 15]. The efficiency of a pair of nodes in a graph is the multiplicative inverse of the shortest path distance between the nodes [13]. The average global efficiency of a graph is the average efficiency of all pairs of nodes. K-means clustering from the python scikit-learn library [16] was used for patient subgroup identification. To assess the robustness of the identified patient subgroups, we did two complementary analyses on the patient cohort: First, silhouette scores were computed from *k* = 2–6 clusters to evaluate optimal cluster number. Second, bootstrap cluster stability at *k* = 2 was assessed using 1000 resampling iterations with replacement, computing the Adjusted Rand Index (ARI) between the reference clustering (*k* = 2) and each bootstrap replicate. The mean ARI and 95% interval were reported.

Lobular thickness, *p* values, patient distributions, and network graph metrics were plotted using the python library matplotlib [17]. The heatmap showing lobular thickness per patient was plotted using the python library PyComplexHeatmap [18]. Connectivity matrices were plotted using the python library graspologic [19]. Visual network representations were plotted using the python libraries netplotbrain [20] and NetworkX.

We used supervised machine learning to classify patient phenotypes (anti-Yo PCD, SCA1) and healthy controls using the extracted cerebellar neuroimaging features. This analysis was done using scikit-learn [16]. Subjects with missing feature data were excluded, and demographic and acquisition metadata (Age, Sex, SNR, mSNR, scale factor, and ICV) were removed from the feature set so that the learned decision rules reflect cerebellar morphometry rather than group demographics. We further pruned the feature set of redundant predictors: The raw cortical-thickness columns correlate very strongly with their scale-factor-normalized counterparts (Pearson *r* = 0.98, min 0.95 across lobules), so we retained only the normalized versions together with the corresponding thickness asymmetries. Volumes (cm^3^ and %), gray-matter measures, and volume asymmetries were retained unchanged. Because cortical thickness declines with age and the three cohorts are not age-matched, each remaining morphometric feature was additionally age-residualized within cross-validation: for every fold, a univariate linear regression of the feature on Age was fit on the training subjects only, and the fitted line was subtracted from both the training and the test values. This removes the linear age component from the features. Then, two complementary classification strategies were applied: First, a univariate analysis identified the ’best single feature’ for each classification task (e.g., PCD vs. other groups) by selecting the residualized feature with the highest mean Area Under the Receiver Operating Characteristic curve (AUC-ROC) from a 5-fold stratified cross-validation. Second, a Random Forest classifier (500 trees, balanced class weights) [21] was trained and evaluated using the same 5-fold stratified cross-validation scheme to capture multivariate feature interactions. This feature’s importance was derived from the mean decrease in Gini impurity [22]. To assess whether the Random Forest classifier performed above chance, we performed label-permutation testing: class labels were randomly shuffled 1000 times and the full CV pipeline was repeated for each permutation with a Random Forest of 100 estimators, and an empirical p value was computed as the proportion of permuted mean AUCs meeting or exceeding the observed mean AUC. Visualizations were generated again with matplotlib [17] and seaborn [23].

## Results

The median age of the patients included in this study was 71±12, 49±16 and 75±4 for PCD, SCA1 and healthy subjects, respectively. The ratio of man/woman per group was 0:1, 1:1, 0:1. The Scale for the Assessment and Rating of Ataxia (SARA) [10] score was 25±10 for the PCD and 23.6±6.7 for the SCA1 patients and the median modified Rankin scale (mRS) [24] in the PCD was of 4 [range 3–5] and non-evaluated in the other groups. The median CAG length was of 47±7 in the SCA1 group.

### Cortical thickness analysis

**Fig. 1:**
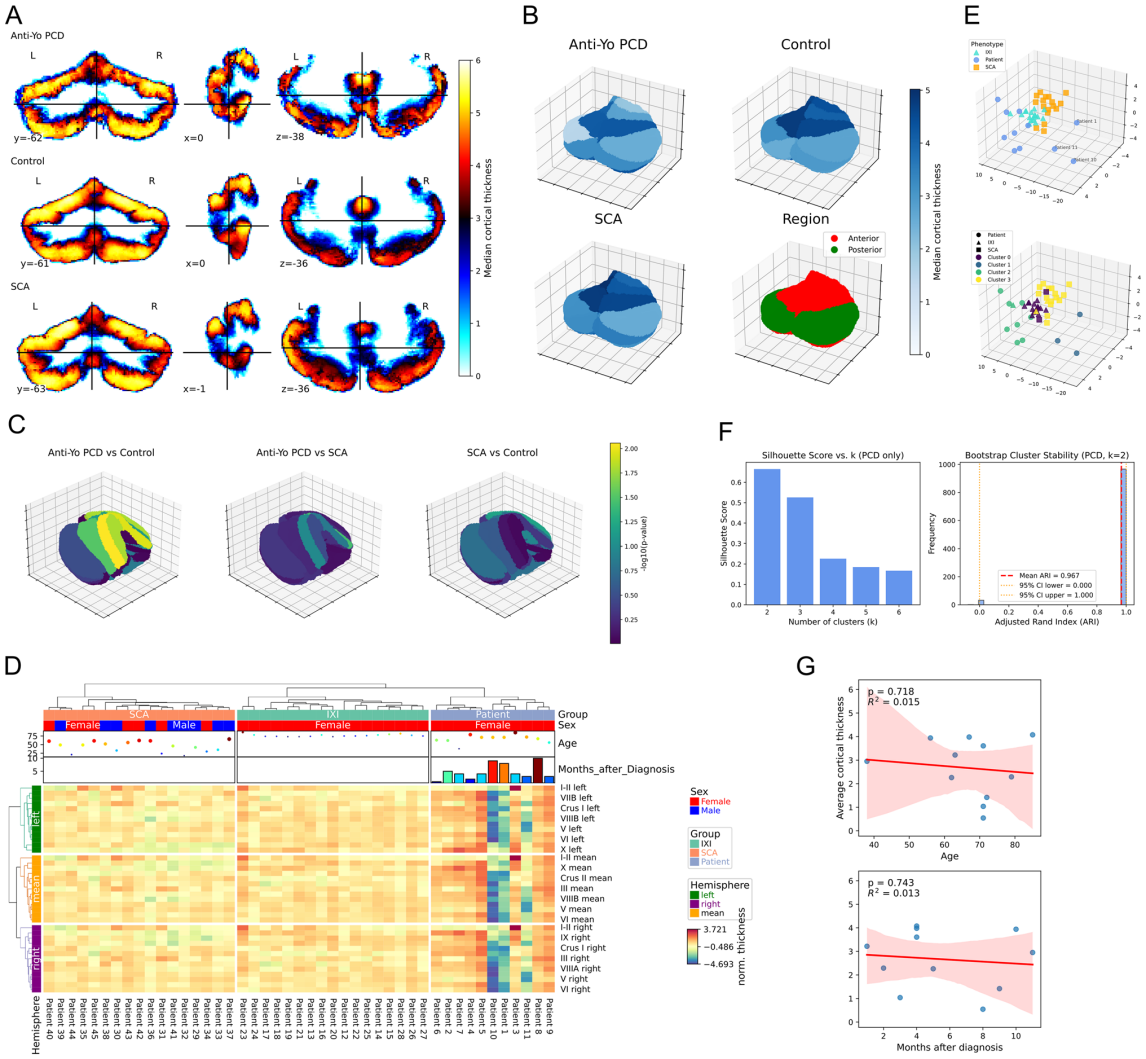
**A** Median cerebellar cortical thickness of control (top), anti-Yo PCD (middle), and SCA1 (bottom) patients in coronal, horizontal, and sagittal view. **B** 3D view of median cortical thickness per cerebellum lobule (left and right hemisphere). Anterior vs. posterior distinction. **C** 3D view of -log10 transformed FDR-corrected *p*-values of multivariate analysis per cerebellum lobule. **D** Heatmap showing normalized thickness for each lobule, hemisphere, and patient, clustered by patient group. **E** 3D PCA representation of all patients, showing patient group clustering into two subgroups. **F** Cluster stability of the two anti-Yo PCD cortical-thickness subgroups. Left: silhouette score for the PCD subjects. Right: distribution of Adjusted Rand Index values across 1000 bootstrap resamples (with replacement) at *k* = 2, compared against the reference clustering of the full PCD sample. **G** Scatter plots showing average cortical thickness against age and months after diagnosis for the anti-Yo PCD group

Analysis of median cortical thickness revealed significant reductions in multiple cerebellar lobules in anti-Yo-positive PCD patients compared to both control and SCA1 groups, with the anterior lobules predominantly affected (Fig. 1a, b, c, d, Table 1). Lobules I–II showed the most pronounced numerical median reduction (−95.6% left, −94.2% right), while controls displayed stable cortical thickness values.

**Table 1:** Pairwise comparisons of cerebellar lobules. For each lobule and hemisphere, the median cortical thickness was calculated across all participants. Differences in thickness were tested for statistical significance using univariate (Welch’s *t* test) and multivariate (linear regression adjusted for age) analyses. The beta values from the regression represent the effect size of group (anti-Yo PCD, control, or SCA1) membership. Significant results are highlighted with an asterisk (∗*p* < 0.05)

Lobule	Median thickness			PCD vs Control			PCD vs SCA			Control vs SCA		
	Control	Patient	SCA	*p*-value univar	Beta	*p*-value multivar	*p*-value univar	Beta	*p*-value multivar	*p*-value univar	Beta	*p*-value multivar
Lobule I–II	1.48	0.06	0.56	0.76	0.05	0.98	0.91	−0.11*	0.86	0.20	−0.49*	0.33
Lobule III	2.06	1.78	1.73	0.92	0.25	0.91	0.57	−0.85*	0.51	0.27	−0.74*	0.11
Lobule IV	4.60	3.12	4.75	0.08	−1.86*	0.01*	0.17	1.63	0.15	0.56	0.21	0.47
Lobule V	4.75	4.04	5.02	0.08	−1.62*	0.01*	0.07	1.67	0.04*	0.20	0.14	0.47
Lobule VI	4.78	4.18	4.78	0.21	−1.15*	0.08	0.28	0.61	0.52	0.70	−0.34*	0.18
Lobule Crus I	3.17	1.46	3.15	0.41	−1.75*	0.02*	0.48	−0.44*	0.69	0.94	−0.81*	0.06
Lobule Crus II	2.55	3.22	3.20	0.62	−0.05*	0.98	0.91	−0.78*	0.51	0.10	−0.23*	0.47
Lobule VIIB	2.77	4.05	3.65	0.21	0.94	0.13	0.91	−0.35*	0.69	0.00*	0.22	0.47
Lobule VIIIA	4.18	4.53	4.16	0.92	0.01*	0.99	0.77	0.13	0.85	0.56	0.03*	0.90
Lobule VIIIB	4.53	4.31	3.24	0.21	−0.95*	0.14	0.77	−0.25*	0.82	0.00*	−0.58*	0.18
Lobule IX	3.08	2.47	1.82	0.59	−0.23*	0.91	0.46	−0.27*	0.80	0.00*	−0.27*	0.47
Lobule X	1.29	0.49	0.55	0.81	−0.28*	0.91	0.57	−0.22*	0.80	0.06	−0.05*	0.89
Lobule I-II (R)	0.88	0.05	0.41	0.77	0.18	0.91	0.91	0.14	0.85	0.27	−0.33*	0.47
Lobule III (R)	1.90	1.02	1.11	0.92	−0.14*	0.91	0.77	−0.93*	0.51	0.27	−0.81*	0.11
Lobule IV (R)	4.40	1.98	3.94	0.09	−1.94*	0.02*	0.28	1.40	0.33	0.27	0.08	0.89
Lobule V (R)	4.05	2.77	4.22	0.08	−2.03*	0.01*	0.07	1.59	0.11	0.52	0.07	0.77
Lobule VI (R)	4.56	4.24	4.58	0.21	−1.37*	0.03*	0.28	0.57	0.57	0.65	−0.26*	0.26
Lobule Crus I (R)	2.74	2.53	2.55	0.92	−0.78*	0.32	0.91	−1.17*	0.46	0.67	−0.63*	0.18
Lobule Crus II (R)	2.98	3.22	3.54	0.92	−0.58*	0.39	0.63	−0.35*	0.69	0.16	−0.16*	0.47
Lobule VIIB (R)	2.88	4.28	3.13	0.58	0.57	0.60	0.91	−0.47*	0.69	0.17	0.06	0.89
Lobule VIIIA (R)	3.97	4.75	3.67	0.87	0.12	0.91	0.51	−0.77*	0.51	0.30	−0.24*	0.47
Lobule VIIIB (R)	4.74	4.57	3.70	0.38	−0.85*	0.13	0.51	−0.84*	0.51	0.01*	−0.69*	0.18
Lobule IX (R)	3.30	3.25	1.67	0.92	0.17	0.91	0.28	−0.60*	0.65	0.01*	−0.19*	0.63
Lobule X (R)	1.05	0.83	0.48	0.92	0.33	0.86	0.28	−0.68*	0.51	0.01*	−0.42*	0.18

Quantitatively, cortical thickness was balanced between the left and right hemispheres across all groups (Fig. 1b, d). In controls and SCA1 patients, minimal inter-individual variability was observed (*ct*_*control*_ = 3.11 ± 0.27, *ct*_*SCA1*_ = 2.83 ± 0.51). However, anti-Yo PCD patients demonstrated substantial heterogeneity (*ct*_*antiYo*_ = 2.67 ± 1.25), indicating variability in disease progression, which prompted the subgroup analysis described below. Importantly, neither age nor time after diagnosis significantly influenced cortical thickness in the anti-Yo group (Fig. 1G and supplementary table 1).

Figure 1A illustrates the median cortical thickness of cerebellar lobules in coronal, horizontal, and sagittal planes for each group. In anti-Yo PCD patients, the largest median-thickness reductions were observed in the most anterior lobules (I–II) although the high inter-individual variance in this region prevented FDR-corrected significance. Lobules IV and V exhibited significant thinning compared to controls, suggesting a progressive anterior-to-posterior gradient of degeneration. Posterior involvement was more selective: Crus I in the anti-Yo PCD group showed a significant reduction relative to controls (*β* = −1.75, FDR-corrected *p* = 0.016), whereas Crus II thickness was not reduced relative to controls in either hemisphere (Table 1). SCA1 patients exhibited a milder, posterior-leaning pattern of cortical-thickness reduction, with lower medians in posterior lobules (Crus I, VIIB, VIIIB and IX) in the univariate analysis, although none of the Control vs. SCA1 differences reached FDR-corrected significance after adjustment for age (Table 1). Control participants displayed homogeneous cortical thickness across all regions, providing a stable baseline for comparison (Table 1).

In Fig. 1C, statistical analysis of the cortical thickness differences (*p* values with False Discovery Rate (FDR) correction [10]) highlights significant reductions in multiple lobules for anti-Yo PCD compared to both controls and SCA1. For example, lobules I–II exhibited significant reductions (*p* = 0.011 left; *β* = −1.94, *p* = 0.016 right), V (*β* = −1.62, *p* = 0.011 left; *β* = −2.03, *p* = 0.0088 right), Crus I left (*β* = −1.75, *p* = 0.016), and VI right (*β* = −1.37, *p* = 0.031) were significantly reduced in the anti-Yo PCD group relative to controls. These results emphasize the importance of anterior lobules as markers for anti-Yo PCD pathology, while highlighting the heterogeneity of involvement at the most anterior pole.

### Correlation and network analysis of lobules

**Fig. 2:**
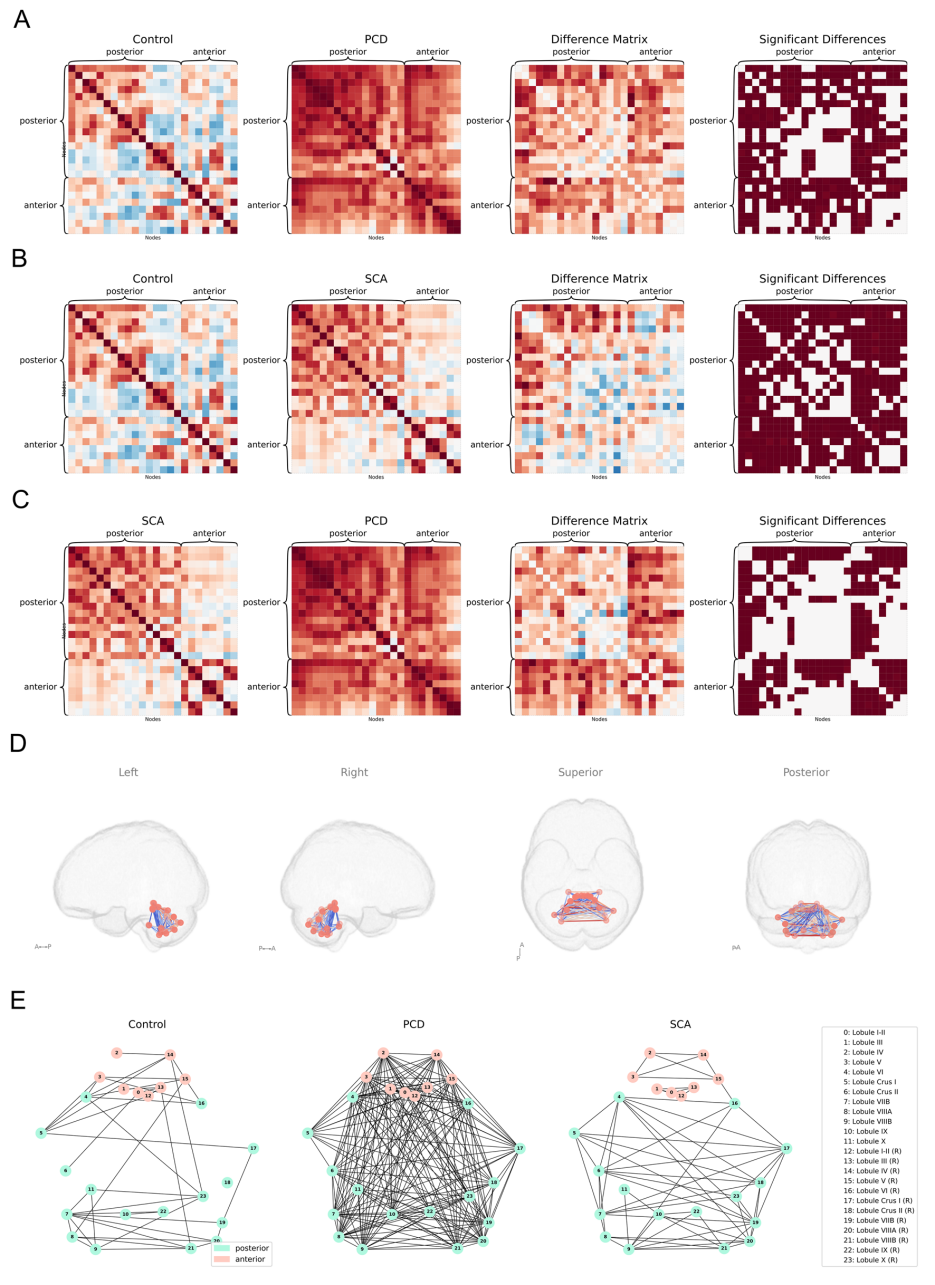
Cortical thickness correlation heatmap of different lobules, separated into anterior and posterior regions for **A** Control and anti-Yo PCD group; **B** Control and SCA1 group; **C** SCA1 and anti-Yo PCD group. **D** Anti-Yo PCD cerebellar lobule network mapped to the brain. Correlation coefficients of lobule pairs were used as edge weights, with a threshold of |r| > 0.5 to determine connections. Red edges represent positive, and blue edges represent negative correlation. The 3D coordinates of the nodes were determined by calculating the center of mass of the corresponding lobules. **E** Network graph of cerebellum lobules for each patient group, with nodes colored for region (anterior/posterior), superior view

Cortical thickness correlations within each group are visualized in Fig. 2A, B, C. Anti-Yo PCD patients demonstrated strong correlations across anterior and posterior lobules, reflecting a network-wide alteration in cerebellar structure. SCA1 patients exhibited high correlations predominantly in posterior lobules, whereas the control group showed weaker and less consistent correlations, especially in anterior lobules.

To better understand these structural relationships, a structural covariance network was constructed using correlation coefficients (threshold |*r*| > 0.5). In the anti-Yo PCD group, the cerebellar network was densely connected, with *n*_*e*_ = 198 edges compared to *n*_*e*_ =42 for controls and *n*_*e*_ = 64 for the SCA1 group (Fig. 2D, E). The high density of connections in anti-Yo PCD reflects an abnormal co-variance pattern across cerebellar lobules, suggesting widespread disruption in structural integrity. Interestingly, the network exhibited a balanced distribution of connections between anterior and posterior lobules, highlighting the widespread impact of the disease, unlike the control and SCA1 groups, which showed more localized and regionalized connections.

### Graph-based metrics

**Fig. 3:**
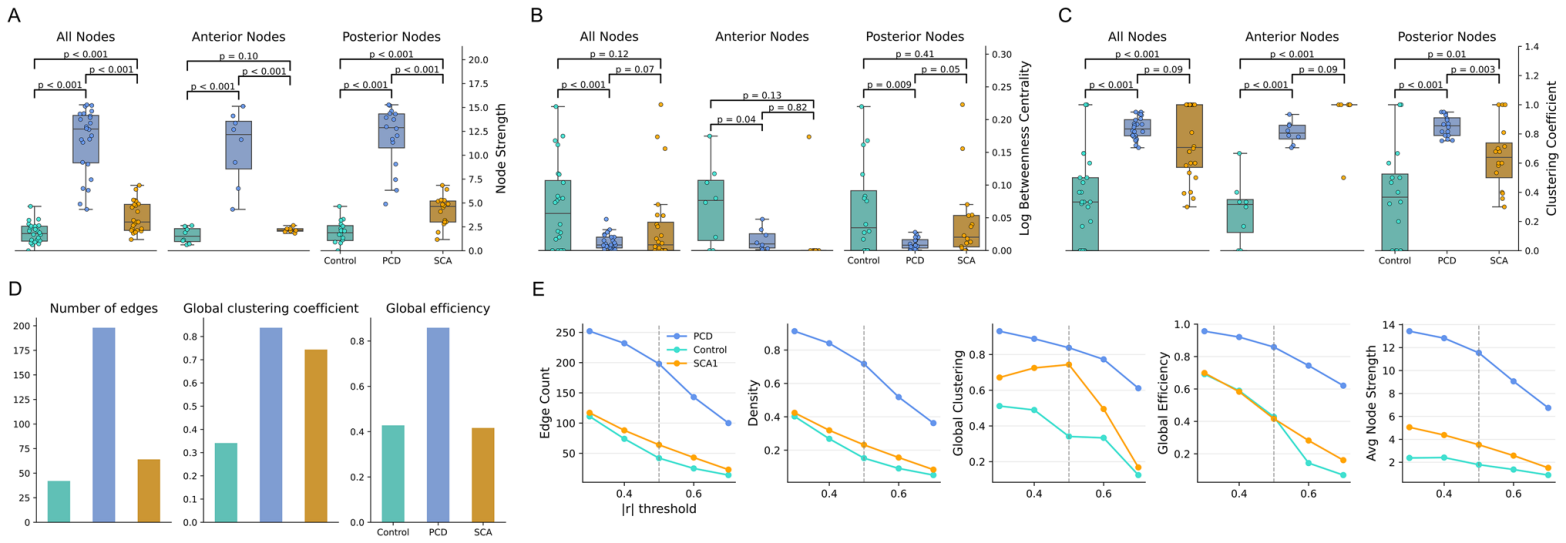
**A**–**C** Comparison of graph network metric distribution between the three groups anti-Yo PCD, control and SCA1 over all nodes, only anterior nodes, and only posterior nodes. **D** Comparison of global network metrics over all three patient groups. **E** Threshold sensitivity analysis for the structural covariance networks. Edge count, graph density, global clustering coefficient, global efficiency, and average node strength are shown for each group across absolute correlation thresholds |*r*| = 0.3–0.7. The ordering of group-level metrics (anti-Yo PCD > SCA1 > Control) is preserved across the tested range for edge count, density, clustering, and node strength

Key network metrics are presented in Fig. 3. Node strength was significantly elevated in the anti-Yo PCD group for both anterior (*p <* 0*.*001) and posterior (*p <* 0*.*001) nodes compared to controls and SCA1 (Fig. 3A). In the SCA1 group, node strength was increased only in posterior nodes (*p <* 0*.*001) relative to controls. This indicates that while SCA1 primarily affects posterior cerebellar regions, anti-Yo PCD induces a more global alteration in cerebellar connectivity.

Betweenness centrality (BC), which measures the influence of a node within the network, was significantly reduced in posterior nodes for SCA1 (*p* = 0*.*041) and in both anterior (*p* = 0*.*03) and posterior nodes (*p* = 0*.*006) for anti-Yo PCD patients (Fig. 3B). This reduction suggests that cerebellar nodes in anti-Yo PCD patients are less pivotal within the network, possibly reflecting an overall degradation in network hierarchy.

Clustering coefficient (CC) was significantly elevated in both anti-Yo PCD and SCA1 groups compared to controls, in anterior (*p <* 0*.*001) and posterior (*p <* 0*.*001) nodes (Fig. 3C). This increased clustering in pathological groups indicates localized strengthening of covariance, which may reflect coordinated co-atrophy of structurally related regions or compensatory mechanisms to preserve functionality.

Global metrics show striking differences between groups: The anti-Yo PCD group exhibited the highest global clustering coefficient (*C* = 0.84) and global efficiency (*E*_*glob*_ = 0.86), indicative of a highly interconnected network. In comparison, controls had the lowest clustering coefficient (*C* = 0.34) and global efficiency (*E*_*glob*_ = 0*.*43), with SCA1 patients displaying intermediate values (*C* = 0*.*74, *E*_*glob*_ = 0*.*42) (Fig. 3D). Sensitivity analysis across absolute correlation thresholds (|*r*| > 0.3–0.7) confirmed that the key network findings (elevated edge count, clustering coefficient, and global efficiency in the PCD group relative to controls and SCA1) were preserved across the tested range (Fig. 3E). For example, at |*r*| > 0.3, the PCD network retained 252 edges versus 111 for controls and 117 for SCA1, and at |*r*| > 0.7, the ordering was preserved (PCD 100 edges, SCA1 23, controls 14). Global clustering coefficient and average node strength followed the same pattern across thresholds. The proportional thresholding analysis produced the same qualitative ordering, supporting the robustness of the reported group differences.

### Subgroup identification in anti-Yo PCD

Clustering analysis within the anti-Yo PCD group identified two subgroups with distinct cortical thickness profiles (Fig. 1B, C). The first subgroup displayed severe atrophy across most lobules, with a mean cortical thickness of 1*.*0±0*.*44. These patients showed widespread network alterations, including elevated clustering coefficients and disrupted global efficiency. The second subgroup resembled the control group, with a mean cortical thickness of 3*.*29±0*.*74, and presented a relatively preserved structural covariance network. Within-PCD silhouette scores across *k* = 2 to 6 reached their maximum at *k* = 2 (silhouette score = 0.67), confirming that two clusters best describe the PCD cortical-thickness structure under this metric. Bootstrap stability (1000 iterations over the 11 PCD subjects) results in a mean ARI of 0.97. However, the 95% confidence interval was wide (0.000–1.000) because a small number of bootstrap samples happened to draw too few distinct subjects to reconstitute two clusters, underscoring the sensitivity of any small-n clustering to the specific resample draw. These results support the existence of a severe atrophy and a near-normal subgroup within the cohort while highlighting that the solution must be interpreted as descriptive rather than as a robust partition. Replication in a larger cohort is essential.

### Classification of patient groups

**Fig. 4:**
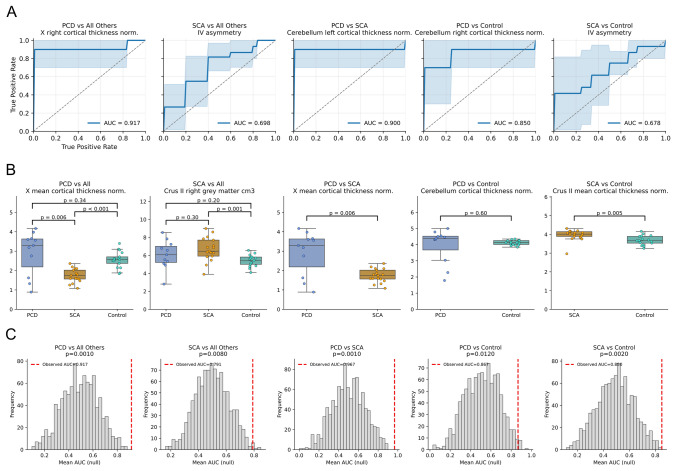
**A** ROC curves for the best-performing single cerebellar feature in distinguishing (from left to right): PCD vs. all, SCA vs. all, PCD vs. SCA, PCD vs. Control, and SCA vs. Control. Each curve shows the mean performance across cross-validation folds, with shaded areas representing the standard deviation. **B** Distribution of best single feature across the relevant patient groups, highlighting the group differences in cerebellar structures. **C** Permutation-test null distributions for the random forest classifier with age-residualized features. For each classification task, histograms show the 1000 mean AUCs obtained after random shuffling of the class labels (age-residualization recomputed per fold); the red dashed line marks the observed (non-permuted) mean AUC

We applied machine learning to the age-residualized cerebellar features to further assess their diagnostic potential. Univariate 5-fold cross-validation identified a best single feature for each classification task. After age-residualization, normalized cortical-thickness measures of lobule X, and cerebellum-level normalized thickness remained the strongest single predictors for anti-Yo PCD versus the other groups (*AUC*_*mean*_ = 0.92 for PCD vs. All Others; *AUC*_*mean*_ = 0.90 for PCD vs. SCA; *AUC*_*mean*_ = 0.85 for PCD vs Control), whereas lobule IV asymmetry emerged as the best single feature for the two SCA1 tasks (*AUC*_*mean*_ = 0.70 for SCA vs. All Others; *AUC*_*mean*_ = 0.68 for SCA vs Control). Both substantially lower than the corresponding PCD-task AUCs, consistent with SCA1’s milder, more posterior atrophy once age-related thinning is removed (Fig. 4 A, B; Table 2).

**Table 2:** Comparison of model performance for patient group classification on pruned, age-residualized cerebellar features. Performance is reported as the mean AUC-ROC (*AUC*_*mean*_), sensitivity, and specificity across five stratified folds. The right-hand block reports the Random Forest classifier (500 trees, balanced class weights) with the empirical p value from a 1000-iteration label-permutation test as the final column

	Best single feature			Random Forest			
Classification task	AUC (± SD)	Sensitivity (± SD)	Specificity (± SD)	AUC (± SD)	Sensitivity (± SD)	Specificity (± SD)	Permutation *p*
PCD vs All others	0.92 (± 0.17)	0.90 (± 0.20)	1.00 (± 0.00)	0.92 (± 0.17)	0.90 (± 0.20)	1.00 (± 0.00)	0.001
SCA vs All others	0.70 (± 0.07)	0.82 (± 0.15)	0.76 (± 0.15)	0.79 (± 0.15)	0.88 (± 0.15)	0.78 (± 0.20)	0.008
PCD vs SCA	0.90 (± 0.20)	0.90 (± 0.20)	1.00 (± 0.00)	0.97 (± 0.07)	1.00 (± 0.00)	0.93 (± 0.13)	0.001
PCD vs Control	0.85 (± 0.20)	0.90 (± 0.20)	0.95 (± 0.10)	0.87 (± 0.11)	0.90 (± 0.20)	0.88 (± 0.15)	0.012
SCA vs Control	0.68 (± 0.23)	0.82 (± 0.15)	0.72 (± 0.27)	0.85 (± 0.19)	0.93 (± 0.13)	0.85 (± 0.20)	0.002

The multivariate random forest classifier (500 trees, balanced class weights) yielded *AUC*_*mean*_ values of 0.97 for PCD vs. SCA, 0.92 for PCD vs. All Others, 0.87 for PCD vs. Control, 0.85 for SCA vs. Control, and 0.79 for SCA vs. All Others on the same age-residualized features (Table 2). Permutation testing with 1000 label shuffles confirmed that all five classification tasks yielded random forest performance above chance: empirical *p* = 0.001 (PCD vs. All Others and PCD vs. SCA), 0.002 (SCA vs. Control), 0.008 (SCA vs. All Others), and 0.012 (PCD vs. Control) (Fig. 4C). The top 5 most influential features per task, derived from the mean decrease in Gini impurity on a whole-sample residualized fit, are detailed in Supplementary Table 2. They consist exclusively of cerebellar morphometric measures, with normalized cortical thickness (anterior and whole-cerebellum) dominating the anti-Yo PCD comparisons and gray-matter volumes of Crus I and lobule IV appearing for the SCA1 comparisons.

## Discussion

Using a multimodal neuroimaging approach, we employed structural MRI, network covariance analysis, and voxel-wise and region-wise quantitative assessments to fully characterize cerebellar structural changes. Our methodology allows for the identification of distinct atrophy profiles, suggesting the existence of two separate patterns of cerebellar degeneration in PCD patients. This dual pattern hypothesis not only enhances our understanding of heterogeneity within PCD but also provides a foundation for future research into targeted therapeutic strategies.

Our study also is in line with previous studies showing that around one-third of anti-Yo PCD harbored cerebellar atrophy at diagnosis, but using semi-quantitative MRI analysis [3]. Indeed, this study confirms these previous results, but future studies should be focused on the validation and longitudinal follow-up of the cerebellar atrophy pattern of these patients and the factors that may modulate or participate in the heterogeneity of the observed atrophy profiles.

The originality of this study lies in the integrated use of diverse quantitative imaging techniques to dissect the cerebellar structural alterations in PCD. While previous research has predominantly focused on qualitative assessments or single-modality analysis, our comprehensive approach offers a more nuanced view of cerebellar pathology. Furthermore, by including both hereditary (SCA1) and control groups, we establish a comparative framework that underscores the unique aspects of PCD-related cerebellar atrophy.

### Morphological patterns of atrophy

A central finding of our study was the distinct pattern of cerebellar atrophy in anti-Yo PCD patients, which predominantly affected the anterior cerebellar lobules. This anterior-predominant thinning, with lobules IV and V showing robust FDR-corrected reductions, and lobules I–II showing the largest absolute median drop despite high inter-individual variance, aligns with prior studies suggesting that anterior cerebellar regions are particularly vulnerable to the neurotoxic effects of anti-Yo antibodies [1]. In contrast, SCA1 patients showed a milder, posterior-leaning trend in cortical thickness, with numerically lower medians in posterior lobules (Crus I, VIIB, VIIIB, and IX) although these Control–SCA1 differences did not reach FDR-corrected significance after age adjustment. This is broadly consistent with the predominantly posterior atrophy profile reported for SCA1, but the modest effect sizes observed here likely reflect the early disease stage of our cohort and the small sample size.

The identification of two subgroups within the anti-Yo cohort, one with severe atrophy and another with near-normal cortical thickness, suggests significant heterogeneity in disease progression or severity. This variability could reflect differences in immune responses, cancer type, or treatment history. Future studies incorporating longitudinal imaging and immunological profiling could clarify these differences between patients and their clinical relevance.

### Relationship between atrophy and clinical phenotype

The pronounced anterior cerebellar atrophy observed in anti-Yo PCD patients likely contributes to their clinical phenotype, characterized by severe ataxia and coordination deficits. The anterior lobules, including I–II and III, are integral to motor coordination, and their degeneration is consistent with the profound motor impairments reported in these patients. Additionally, the global symmetry of atrophy across hemispheres, as revealed in our analysis, supports the hypothesis of a systemic immune-mediated mechanism targeting the cerebellum rather than lateralized pathology.

In contrast, the posterior cerebellar atrophy observed in SCA1 patients may be more closely associated with non-motor symptoms, such as cognitive and affective disturbances, which are increasingly recognized as key features of spinocerebellar degeneration [25]. These findings highlight the potential utility of regional atrophy patterns as biomarkers for distinguishing between these cerebellar disorders and tailoring clinical management strategies.

### Insights from structural covariance network analysis

Advanced MRI analysis concepts, such as SCN, have gained widespread popularity in neuroscience and neurology due to their ability to measure morphometric covariance (similarity) of brain regions in a non-invasive manner [26, 27]. These networks can be constructed using various morphometric measures, including gray matter volume (GMV), cortical surface area, cortical thickness, and cortical gyrification [28–30]. Our findings contribute to this body of knowledge by providing evidence for distinct atrophy patterns, potentially linked to different immunopathogenic mechanisms in PCD.

SCN analysis provided novel insights into the organization and disruption of cerebellar connectivity in anti-Yo PCD and SCA1 patients. In the anti-Yo PCD group, the network was characterized by an abnormally high density of connections, with a significantly elevated number of edges compared to both controls and SCA1. This dense covariance, reflected in the heightened global clustering coefficient and efficiency, could arise from several non-exclusive mechanisms. One possibility is compensatory reorganization attempting to preserve cerebellar functionality. However, alternative explanations should be considered. The heightened covariance could also reflect coordinated co-atrophy across structurally related regions, a methodological artifact of reduced between-subject variance in the anti-Yo PCD group (where most subjects show severe atrophy), or inflated correlations due to the small sample size. At the same time, the observed reduction in betweenness centrality suggests a loss of hierarchical structure within the network (Fig. 2, 3).

The balanced distribution of anterior and posterior connections in the anti-Yo PCD group further underscores the systemic nature of the disease, implicating widespread disruption in cerebellar architecture rather than localized damage. Such patterns may reflect the broad targeting of Purkinje cells by anti-Yo antibodies, as these cells form a critical component of cerebellar circuitry.

In SCA1 patients, the structural covariance network exhibited more localized alterations, with a specific emphasis on posterior regions. Posterior lobules, such as Crus I and VIIIA, showed increased clustering coefficients, suggesting regionally strengthened connectivity, possibly reflecting localized pathological clustering. However, the reduced global efficiency in the SCA1 group indicates impaired long-range connectivity, consistent with the regionalized degeneration characteristic of hereditary ataxias. These findings align with existing literatures on SCA1, where posterior cerebellar regions are disproportionately affected and connectivity deficits parallel the progression of clinical symptoms [31–33].

Overall, the SCN analysis in this study provides a framework for understanding how regional morphological changes translate into network-level disruptions. By integrating these findings with clinical and functional data, future studies could further elucidate the relationship between connectivity patterns and symptom severity, enabling the development of targeted therapeutic interventions.

### Comparison with other neurological diseases

The observed patterns of cerebellar atrophy and network disruptions in anti-Yo PCD and SCA1 patients share similarities with other neurological diseases, highlighting common mechanisms underlying cerebellar degeneration. For example, longitudinal volumetric MRI has shown significant cerebellar cortical volume loss in anti-*N*-methyl-D-aspartate receptor (anti-NMDAR) encephalitis, and the degree of cerebellar atrophy at two-year follow-up correlates with clinical outcome [34]. Although the distribution of atrophy within the cerebellum was not mapped in that study, the result supports the idea that antibody-mediated neuro-inflammation can lead to progressive cerebellar degeneration. In other neuroimmunological conditions, such as multiple sclerosis (MS), cerebellar atrophy has been associated with depression and fatigue, suggesting a neural substrate for these symptoms. This relationship, partly linked to cerebellar atrophy [35], implies that cognitive dysfunction may arise from altered brain connectivity in these regions. Interestingly, in another recent study that analyzed common neuroinflammatory diseases, patients with AQP4+ neuromyelitis optica spectrum disorder (NMOSD) showed widespread GM cerebellar atrophy [36]. This reinforces the idea that quantitative cerebellum atrophy studies could be used in a broad range of neurological conditions.

In SCA1, the posterior-predominant atrophy and the localized network alterations resemble patterns seen in Alzheimer’s disease (AD), where the posterior cortical regions are disproportionately affected [37, 38] that are hypothesized to be linked with “dysmetria of thought” [39]. It should be noted that other studies focused on voxel-based-morphometry and volumetry have identified a prominent atrophy in the cerebellar white matter and in other structures of the central nervous system (CNS), like brainstem and basal ganglia [4, 32, 40, 41]. Similar to SCA1, AD is characterized by regionally specific atrophy that disrupts long-range connectivity while preserving local clustering. These parallels suggest that shared principles of network disruption—such as the preferential vulnerability of posterior regions—may underlie diverse neurodegenerative disorders. Cortex and cerebellum are densely interconnected through reciprocal input and output projections that form segregated circuits. For instance, lobules Crus I and II are strongly linked to prefrontal regions [42, 43]. Exploring these commonalities could provide insights into the mechanisms driving selective regional vulnerability in the cerebellum and other brain regions. Future studies considering the full brain-cerebellum networks in larger series could further explore these relationships in these conditions.

Our findings also have implications for understanding the broader role of the cerebellum in neurological diseases. Increasing evidence links cerebellar dysfunction to a range of non-motor symptoms, including cognitive and affective disturbances [25]. The structural and network alterations identified in this study could help clarify how cerebellar degeneration contributes to these symptoms, both in SCA1 and other conditions such as multiple sclerosis, where cerebellar involvement is not considered a key feature. However, it is important to bear in mind that many previous studies excluded the cerebellum, and software packages remove the cerebellum from consideration.

By comparing the structural and network changes observed in anti-Yo PCD and SCA1 to those in other diseases, our study highlights both shared and distinct features of cerebellar pathology. These comparisons underscore the value of multimodal imaging approaches in unraveling the complexities of cerebellar degeneration and pave the way for cross-disease investigations that could inform diagnosis and treatment strategies across a wide spectrum of neurological disorders.

### Diagnostic value of cerebellar features

In addition to regional and network-level analyses, we applied machine learning techniques to assess the diagnostic potential of cerebellar features. Both univariate (best single feature) and multivariate (Random Forest) approaches achieved promising AUC values in 5-fold cross-validation on a pruned, age-residualized cerebellar feature set (Table 2). Permutation testing with 1000 label shuffles confirmed that all five classification tasks yielded statistically significant random forest performance above chance (*p* ≤ 0.012), strengthening the validity of our results. However, there are several caveats: The small sample size makes these classifiers prone to overfitting. The absence of an external validation cohorts means that the reported AUCs should be considered as optimistic upper bounds. Age-residualization substantially reduced the random forest AUC for SCA vs. Control while leaving the PCD tasks comparatively unchanged, indicating that the PCD signal is driven primarily by disease-related morphometric differences rather than age-related variance. These results should be viewed as hypothesis-generating rather than definitive evidence for diagnostic classifiers; independent replication in larger, multi-center cohorts is essential before any clinical translation.

### Limitations and future directions

While our study provides novel insights, several limitations must be acknowledged. First, our analysis was restricted to cross-sectional MRI data obtained at diagnosis. As such, we could not assess longitudinal changes in atrophy or their relationship with disease progression and treatment. Future studies incorporating serial imaging could elucidate the temporal dynamics of cerebellar degeneration and identify early markers of disease progression.

The identification of two subgroups within the anti-Yo PCD cohort via K-means clustering should be interpreted cautiously. With only 11 PCD subjects, the influence of any single case on the clustering solution is substantial. Whether these subgroups reflect genuine biological subtypes, staging of disease progression, or sampling variability cannot be resolved with the current data.

Expanding the cohort and incorporating multi-center datasets would enhance statistical power and robustness. Third, our analysis focused on structural MRI metrics and did not integrate functional or metabolic imaging modalities, which could provide complementary insights into cerebellar dysfunction. Fourth, the different distribution of sex per disease (i.e., anti-Yo PCD only represented in females), could have some impact on the results. Another notable limitation concerns scanner heterogeneity: data were acquired across different scanners (1.5 T and 3 T) from different vendors, and healthy controls were drawn from the external IXI dataset. Per-subject scanner metadata were not available for all participants, precluding formal statistical harmonization (e.g., ComBat) or the inclusion of scanner field strength as a covariate. Residual scanner effects may therefore be present in the data and represent an important potential confound, particularly for the structural covariance network analysis.

The substantial age differences between groups (anti-Yo PCD: 71 ± 12 years; SCA1: 49 ± 16 years; IXI controls: 75 ± 4 years) also represent an important potential confound. Although age was included as a covariate in all multivariate cortical-thickness models, statistical adjustment may not fully eliminate age-related effects on cortical thickness, particularly given the large age range and small sample sizes. The two-layer mitigation of our patient group classification (removing demographic/acquisition metadata and age-residualizing remaining morphometric feature) removes both age as an explicit predictor and the linear age component of the features themselves. However, non-linear age-by-disease interactions remain unmodeled and residual confounding by age cannot be fully excluded. Comparisons across the wide age range should therefore be interpreted cautiously.

Finally, the clinical implications of our findings require further exploration. Linking specific atrophy and network patterns to clinical outcomes, such as motor and cognitive performance, could refine their diagnostic and prognostic utility. Integrating these imaging biomarkers with immunological and molecular data would enable a more comprehensive understanding of disease mechanisms and therapeutic targets.

## Conclusion and perspectives

This study highlights the distinct morphological and network alterations underlying cerebellar degeneration in anti-Yo PCD and SCA1. The identification of anterior-predominant atrophy and network disruptions in anti-Yo PCD advances our understanding of this rare paraneoplastic syndrome and its differentiation from other cerebellar disorders. The use of SCN analysis adds a novel dimension to cerebellar imaging studies, offering insights into the connectivity disruptions that accompany morphological changes.

Future research should prioritize longitudinal imaging and multimodal analyses to fully capture the complexity of cerebellar degeneration in these and related disorders. By integrating advanced imaging techniques with clinical, immunological, and genetic data, we can move closer to personalized diagnostic and therapeutic strategies for patients with cerebellar diseases.

## Supplementary Information

Below is the link to the electronic supplementary material.

Supplementary file1 (XLSX 15 KB)

## Data Availability

The dataset is available under 10.5281/zenodo.16094263. All code used for data analysis and figure generation is available at: https://github.com/tamelenak/pcd-spatial-analysis.
